# Supplementary figures and images for: Tracing the Repertoire of Promiscuous Enzymes along the Metabolic Pathways in Archaeal Organisms
Source: Life (Basel). 2017 Jul 13;7(3):30. doi: 10.3390/life7030030 (PMC5617955; doi:10.3390/life7030030)

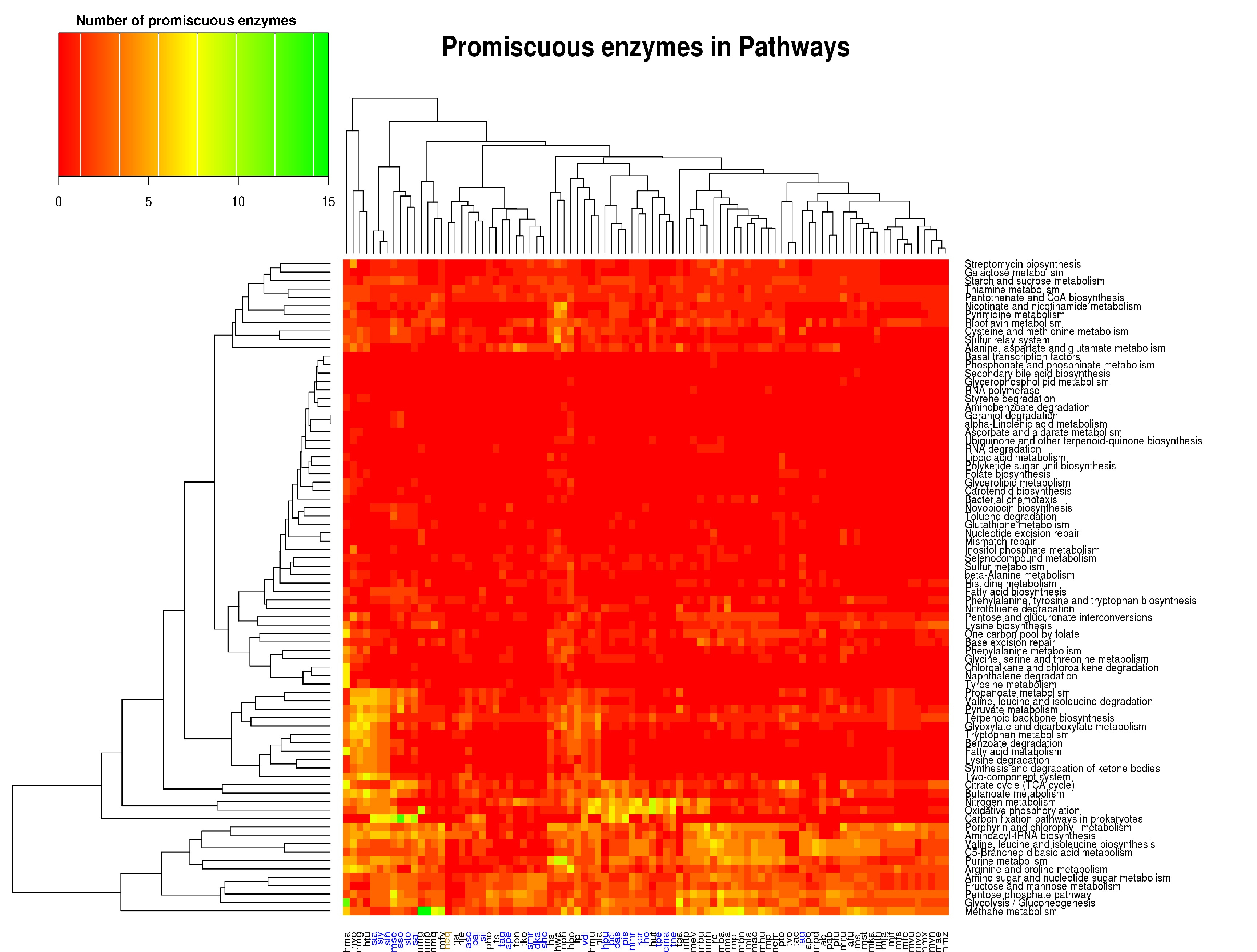

Supplement: Supplementary file 1 [file life-07-00030-s001.zip › Figure_S1.jpg]
