# Supplementary material for: Tracing the Repertoire of Promiscuous Enzymes along the Metabolic Pathways in Archaeal Organisms
Source: Life (Basel). 2017 Jul 13;7(3):30. doi: 10.3390/life7030030 (PMC5617955; doi:10.3390/life7030030)
Supplement: Supplementary file 1 [file life-07-00030-s001.zip › Figure_S1.docx]

**Figure S1. Heatmaps of distribution of promiscuous enzymes in KEGG metabolic pathways.** Heatmaps was elaborate with ggplot package of R, and the clustering groups were made with the complete linkage method and use Euclidean distance measure. The identifiers of organisms according to KEGG are shown. In black color the organisms of the phylum Euryarchaeota; in blue the Crenarchaeota; and in golden Nanoarchaeota.
